# Risk of Ischemic Stroke, Hemorrhagic Stroke, and All-Cause Mortality in Retinal Vein Occlusion: A Nationwide Population-Based Cohort Study

**DOI:** 10.1155/2018/8629429

**Published:** 2018-09-09

**Authors:** Yu-Yen Chen, Yung-Feng Yen, Jun-Xian Lin, Shih-Chao Feng, Li-Chen Wei, Yun-Ju Lai, Ying-Cheng Shen

**Affiliations:** ^1^Department of Ophthalmology, Taichung Veterans General Hospital, Taichung 407, Taiwan; ^2^School of Medicine, National Yang-Ming University, Taipei 112, Taiwan; ^3^Community Medicine Research Center and Institute of Public Health, National Yang-Ming University, Taipei 112, Taiwan; ^4^Section of Infectious Diseases, Taipei City Hospital, Taipei 111, Taiwan; ^5^Department of Health Care Management, National Taipei University of Nursing and Health Sciences, Taipei 112, Taiwan; ^6^Division of Endocrinology and Metabolism, Department of Internal Medicine, Puli Branch of Taichung Veterans General Hospital, Nantou County 545, Taiwan; ^7^Department of Exercise Health Science, National Taiwan University of Sport, Taichung 404, Taiwan

## Abstract

**Purpose:**

To investigate whether the risk of subsequent stroke, ischemic stroke, hemorrhagic stroke, and all-cause mortality is increased among retinal vein occlusion (RVO) patients compared to non-RVO patients.

**Methods:**

From the entire population of the Taiwan National Health Insurance Research Database (NHIRD) from 2001 to 2013, a total of 22919 subjects with RVO were enrolled in the RVO group, and 114595 propensity score (PS)-matched non-RVOs were enrolled in the comparison group. PS matching was based on age, gender, obesity, diabetes, hypertension, hyperlipidemia, coronary artery disease, atrial fibrillation, hyperviscosity syndrome, Charlson comorbidity index, glaucoma, and the use of antithrombotic drugs. A multivariate Cox regression analysis was used to estimate the adjusted hazard ratios (HRs) with a 95% confidence interval (CI) for each of the clinical outcomes, including stroke, ischemic stroke, hemorrhagic stroke, and all-cause mortality. Furthermore, we divided the RVO group into the branch retinal vein occlusion (BRVO) group and the central retinal vein occlusion (CRVO) group and separately compared their subsequent risks of the clinical outcomes with those of the comparison group.

**Results:**

After adjusting for PS, the RVO group had a significantly higher risk of stroke (adjusted HR = 1.36; 95% CI: 1.32–1.40), ischemic stroke (adjusted HR = 1.36; 95% CI: 1.32–1.40), and hemorrhagic stroke (adjusted HR = 1.34; 95% CI: 1.24–1.44). However, the all-cause mortality did not exhibit significant differences. Furthermore, both the BRVOs and CRVOs had a significantly higher risk of subsequent stroke, ischemic stroke, and hemorrhagic stroke than did the comparisons, whereas all-cause mortality was similar among the groups.

**Conclusions:**

People with RVO are at a significantly greater risk of developing stroke, ischemic stroke, and hemorrhagic stroke. However, RVO does not significantly increase the risk of all-cause mortality.

## 1. Introduction

Retinal vein occlusion (RVO) is the second most common retinal vascular disease. It results from thrombosis of the retinal vein due to external compression by an atherosclerotic artery or increased blood viscosity [1, 2]. Depending on the site of occlusion, RVO can be classified as either branch RVO (BRVO) or central RVO (CRVO). The risk factors for RVO include hypertension, diabetes, hyperlipidemia, arteriosclerosis, and older age [3–9]. These are also risk factors for stroke or mortality. In addition, previous studies have revealed that changes in the retinal vessels may reflect similar changes in the cerebral vessels [10, 11]. Therefore, it is important to investigate the relationship between RVO and the subsequent risk of stroke or mortality.

Previous population-based studies on the association between RVO and stroke revealed conflicting findings. Studies in the USA, Denmark, and Korea revealed a significantly higher risk of stroke among RVO patients [3,12–14]. However, Ho et al. utilized the Taiwan National Health Insurance Research Database (NHIRD) and found no significant association between RVO and subsequent stroke, except in the 60- to 69-year group [15]. This may have been due to insufficient statistical power because they used a sampled database and not the entire population database. A truly statistically significant trend might be masked when the number of cases is insufficient. Besides, the inconsistent results of previous studies may have been due to discrepancies in the inclusion criteria (e.g., some studies did not exclude patients with previous stroke) or to differences in the follow-up duration (e.g., a wide variation between 1.5 and 12 years). Furthermore, most previous studies evaluated the outcome of overall stroke and did not focus specifically on ischemic stroke or hemorrhagic stroke. Since ischemic and hemorrhagic stroke have different clinical patterns (clot vs. bleeding) and may have different relevance for RVO, it would be more appropriate to analyze the two types of stroke separately. Therefore, in our study, we used the NHIRD with the entire population in Taiwan to include a sufficient number of RVO patients. We excluded patients who had a history of previous stroke to derive a clearer relationship between RVO and new-onset stroke. Additionally, we analyzed data over a long span of 13 years to investigate the long-term effects. Furthermore, we evaluated ischemic stroke and hemorrhagic stroke separately.

Another outcome variable that we wanted to explore was all-cause mortality. Because of its association with serious systemic disorders, RVO seems likely to increase the mortality. However, most follow-up studies found no overall increased risk of mortality [3,13,16–18]. In the Beijing Eye Study, the RVO was significantly associated with an increased mortality rate [19]. These conflicting results should be explored. Therefore, in our study, we further investigated the association between RVO and all-cause mortality.

## 2. Materials and Methods

### 2.1. Study Setting

Taiwan's National Health Insurance (NHI) program was launched in 1995 and currently covers 99% of Taiwan's 23 million residents. The National Health Insurance Research Database (NHIRD) is maintained by the National Health Research Institutes of Taiwan. It contains comprehensive medical records, including outpatient, inpatient, emergency, dental, and traditional Chinese medicine services as well as prescription, procedures, surgeries, vital status and diagnoses, which are registered using the International Classification of Diseases, Ninth Revision, Clinical Modification (ICD-9-CM) codes.

The identification of all patients in the database is encrypted before the data are released. In our study, the clinical outcomes of our interests are stroke, ischemic stroke, hemorrhagic stroke, and all-cause mortality. Based on the healthcare claims of the entire population, we sought to compare the incidence density and risk of each clinical outcome in subjects with and without RVO during the 13-year period. This study was approved by the ethical committee of Yang-Ming University Hospital (2015A018), and the need for written informed consent was waived.

### 2.2. Inclusion and Exclusion Criteria

Using the Taiwan NHIRD, we performed a retrospective cohort study with a study period from January 1, 2001, to December 31, 2013. We first selected patients with RVO who were identified on the claims records. The diagnostic codes of RVO included BRVO (ICD-9-CM code 362.36) and CRVO (ICD-9-CM code 362.35). Patients with RVO diagnoses from January 1, 1995, to December 31, 2000, were excluded to eliminate patients with previous and chronic RVO. The date of the first RVO claim was defined as the index date. We also identified individuals who had never received a diagnosis of RVO as a comparison group. Two comparison cohorts were derived. One was through simple random sampling of the non-RVOs to achieve a sample size 5-fold that of the RVO group without matching the characteristics of the RVO group. This comparison group reflected the real-world conditions. The other type of comparison cohort was the propensity score (PS)-matched cohort. In this situation, the RVO and comparison groups were 1 : 5 matched using the PS matching method [20] for age, gender, index year (the year of the index date in the RVOs and the enrollment date in the comparisons), use of antithrombotic drugs, obesity, diabetes, hypertension, hyperlipidemia, coronary artery disease, atrial fibrillation, chronic kidney disease, hyperviscosity syndrome, Charlson comorbidity index (CCI), and glaucoma. CCI was calculated from the severity of systemic diseases and represented the overall health status [21]. The RVO and comparison groups were tracked during the study period to identify the occurrence of clinical outcomes (stroke, ischemic stroke, hemorrhagic stroke, and all-cause mortality). To ensure that the clinical outcomes were newly diagnosed, patients who received their diagnosis prior to the index date/enrollment date were excluded. For example, when analyzing the risk of ischemic stroke after RVO diagnosis, we excluded patients who had been diagnosed with ischemic stroke before the index/enrollment date.

### 2.3. Statistical Analysis

The demographic/clinical characteristics of the RVO group were compared to those of both comparison groups using a chi-square test for categorical variables and a two-sample *t*-test for continuous variables. The Kaplan–Meier curve with a log-rank test was performed to describe and compare the cumulative hazard curves of stroke, ischemic stroke, and hemorrhagic stroke between the RVO and PS-matched comparison groups. The incidence rate (incidence density, per 10000 years) of each clinical outcome was calculated in the RVO and PS-matched comparison groups. A Cox proportional hazard model was used to estimate the adjusted hazard ratios (HRs) for the occurrence of the clinical outcomes after adjusting for PS. Thereafter, stratified analyses for different age subgroups were performed. Furthermore, we divided the RVO group into the BRVO and CRVO groups and separately compared the HRs for the clinical outcomes among the BRVO, CRVO, and comparison groups after adjusting for PS. All statistical operations were performed using the SAS statistical package, version 9.3 (SAS Institute, Cary, NC, USA).

## 3. Results

### 3.1. The Study Population

This study included 22,919 RVO patients and two comparison groups (each with 114595 non-RVOs).  Table 1 shows the comparison of the characteristics between the RVO group and each of the comparison groups. In the real-world setting, the mean age of the RVO patients was 61.8 years, which was significantly older than the 43.2 years observed for the comparison group (*p* < 0.0001). A greater proportion of people used antithrombotic drugs in the RVO group than in the real-world comparison group (28.1% vs. 7.1%, *p* < 0.0001). RVO patients were observed to more frequently have obesity (2.9% vs. 2.1%), diabetes (37.7% vs. 12.6%), hypertension (78.2% vs. 25.2%), hyperlipidemia (48.0% vs. 19.8%), coronary artery disease (35.7% vs. 10.7%), atrial fibrillation (4.2% vs. 1.0%), chronic kidney disease (11.9% vs. 2.2%), and hyperviscosity syndrome (1.1% vs. 0.3%) compared with the real-world comparison group (all *p* < 0.0001). Furthermore, the prevalence of glaucoma was significantly higher in the RVO group. After PS matching for these variables and for age and gender, these variables were found to be well balanced between the RVO and PS-matched comparison groups. Regarding the outcome of stroke or ischemic stroke, the mean follow-up time in the RVO group was significantly shorter than that in the PS-matched comparison group. However, when the outcome variable was hemorrhagic stroke or all-cause mortality, the two groups had similar mean follow-up periods. Comparing the subsequent occurrence of the clinical outcomes in the RVO group with the PS-matched comparison group during the follow-up period, we identified a significantly higher cumulative incidence in stroke (23.9% vs. 19.4%, *p* < 0.0001), ischemic stroke (21.2% vs. 17.2%, *p* < 0.0001), and hemorrhagic stroke (3.5% vs. 2.7%, *p* < 0.0001). However, the cumulative incidences of all-cause mortality were similar between RVO and comparison groups (13.6% vs. 13.2%, *p*=0.11).

### 3.2. Cumulative Hazard Curves Generated Using the Kaplan–Meier Method

**Figure 1:**
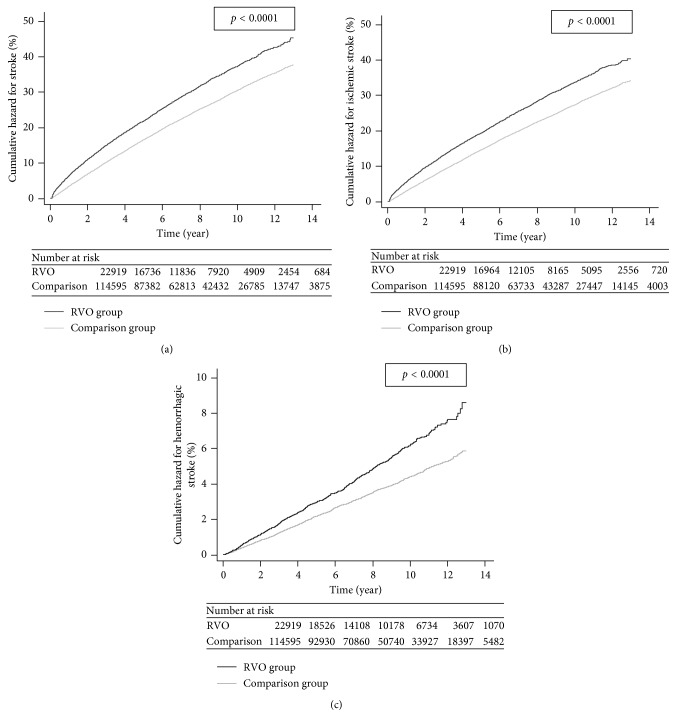
Kaplan–Meier curves for (a) stroke, (b) ischemic stroke, and (c) hemorrhagic stroke in the RVO and the PS-matched comparison groups. The black line represents the RVO group, and the gray line represents the comparison group. RVO: retinal vein occlusion.

Figure 1 illustrates the cumulative hazard curves of stroke, ischemic stroke, and hemorrhagic stroke that were obtained using the Kaplan–Meier method to describe the stratified time-to-event data between the RVO cohort and the PS-matched comparison cohort. In each set shown in the figure, these two curves moved away from each other from the very beginning until the end of the follow-up period. According to the log-rank test, the RVO group had a significantly higher cumulative hazard for stroke, ischemic stroke, and hemorrhagic stroke than did the PS-matched comparison groups (all *p* < 0.0001).

### 3.3. HRs for the Clinical Outcomes Analyzed Using Cox Models

Table 2 displays the incidence density and HRs for the clinical outcomes during the 13-year study period. The incidence densities of stroke, ischemic stroke, hemorrhagic stroke, and all-cause mortality were 494.7, 430.6, and 62.2 per 10000 years in the RVO group, which was higher than the corresponding values of 361.6, 317.0, and 46.6 per 10000 years in the PS-matched comparison group (all *p* < 0.0001). However, the incidence density of all-cause mortality in the RVO group was not significantly higher than that in the comparison group (233.7 vs. 226.6 per 10000 years, *p*=0.11). A Cox regression analysis revealed that after adjusting for PS, the hazards of stroke, ischemic stroke, and hemorrhagic stroke were significantly higher in the RVO group than in the comparison group (HR = 1.37, 1.36, 1.34, respectively; all *p* < 0.0001). Nevertheless, the hazard of all-cause mortality did not reveal a significant difference between the RVO group and the comparison group.

### 3.4. Risk of Clinical Outcomes in Different Age Subgroups

Table 3 shows the stratified analysis of age subgroups regarding the incidence densities and HRs for the clinical outcomes. The statistical significances in the subgroup analysis did not change compared to the previous total cohort analysis. In each age subgroup, the incidence densities of stroke, ischemic stroke, and hemorrhagic stroke were significantly higher in the RVOs than in the comparisons (all *p* < 0.0001). This was also true for the HRs. However, in each age subgroup, all-cause comorbidity was similar in the RVOs and comparisons.

### 3.5. Comparison of the Risks of Clinical Outcomes between the BRVO and CRVO Groups

**Figure 2:**
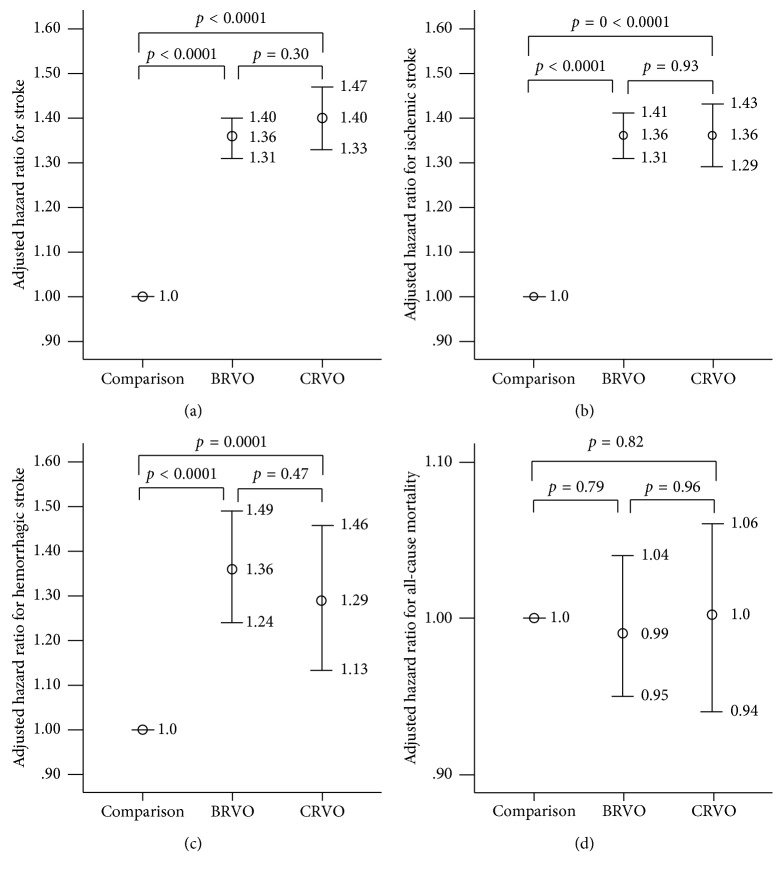
Adjusted hazard ratios for (a) stroke, (b) ischemic stroke, (c) hemorrhagic stroke, and (d) all-cause mortality in the BRVO and CRVO groups compared with the comparison group. BRVO: branch retinal vein occlusion; CRVO: central retinal vein occlusion.

In Figure 2, we divided the RVO group into BRVO and CRVO groups and assessed their risks of developing the clinical outcomes. The clinical outcomes did not have significant differences between the 15392 BRVO patients and the 7527 CRVO patients. When the BRVOs and CRVOs were separately compared with the comparison group, the HRs for stroke, ischemic stroke, and hemorrhagic stroke were all found to be significantly higher after adjusting for PS. However, all-cause mortality was still similar among the groups.

## 4. Discussion

In this 13-year population-based study using complete population data from the Taiwan NHIRD, patients with RVO had a significantly higher risk of developing stroke (adjusted HR = 1.37, 95% CI: 1.33–1.41), ischemic stroke (adjusted HR = 1.36, 95% CI: 1.32–1.40), and hemorrhagic stroke (adjusted HR = 1.34, 95% CI: 1.24–1.44). However, the risk of all-cause mortality was similar in RVOs and non-RVOs (adjusted HR = 1.03, 95% CI: 0.99–1.07). When BRVOs and CRVOs were compared separately with the non-RVOs in the risk of clinical outcomes (stroke, ischemic stroke, hemorrhagic stroke, and all-cause mortality), the patterns of significance remained.

As shown in Table 1, RVO was more prevalent in specific systemic diseases than it was in the real-world non-RVO group. Considering the previous literature, the group differences between the RVOs and non-RVOs were also found to be significant in these diseases, including obesity [22], diabetes [14, 17, 22], hypertension [14, 17, 22, 23], hyperlipidemia [7, 12, 15, 22], coronary artery disease [7, 23], atrial fibrillation [7], chronic kidney disease [7, 14, 22], and hyperviscosity syndrome [22]. In our study, the RVO group had a higher severity of these comorbidities and a higher prevalence of glaucoma. These factors (comorbidities, CCI, and glaucoma) also increased the risk of stroke [24, 25] and are therefore confounders that should be controlled when we explore the impact of RVO on stroke. One strength of our study is that it utilized the PS matching method to deal with the confounding effects and selection bias due to differences in baseline characteristics and medications (antithrombotic drugs); thus, these confounders will be balanced between the RVO and PS-matched cohorts. Thereafter, the incidence rate and the subsequent risk of stroke or mortality could be fairly compared between the groups.

As shown in Table 1 and Figure 1, the cumulative incidence and cumulative hazard of stroke, ischemic stroke, and hemorrhagic stroke were all significantly higher than those of the PS-matched comparison group. It is another strength of our study that we assessed stroke separately as ischemic stroke and hemorrhagic stroke. Because thrombosis in RVO is possibly more closely associated with the development of thrombosis/emboli in ischemic stroke, it is more reasonable to evaluate ischemic stroke and hemorrhagic stroke separately. Only one previous study evaluated ischemic stroke and hemorrhagic stroke separately [14]. In that study, Rim et al. utilized the Korean National Health Research Database and found that RVO significantly increased the risk of ischemic stroke, consistent with the results of our study. However, their study did not reveal a significant group difference in hemorrhagic stroke between the RVOs and their comparisons. This may have been due to an insufficient power to detect a significance in the association (e.g., only 28 of the 1031 RVOs developed hemorrhagic stroke). Another strength of our study was the utilization of the comprehensive, whole population NHIRD in Taiwan, with a long study period of 13 years. With a much larger sample size and a much longer follow-up period, our study provided sufficient power to confirm that the RVO patients had a significantly higher risk of developing not only overall stroke but also ischemic stroke and hemorrhagic stroke.

Regarding all-cause mortality, no difference was found between the RVOs and the comparisons in our study, as shown in Table 2. Our results were not compatible with those of the Beijing Eye Study [19], which showed a significant association between RVO and all-cause mortality among those younger than 70 years. The Beijing Eye Study did not adjust for the impact of systemic diseases, so it is possible that systemic diseases, not RVO itself, were the main causative factors for the higher mortality. In our study, we carefully controlled for systemic diseases and other confounders, suggesting that RVO was not an independent risk factor for all-cause mortality.

Moreover, we conducted stratified analyses according to different age subgroups, as shown in Table 3. The group differences in stroke, ischemic stroke, and hemorrhagic stroke were all significant between the RVOs and comparisons in every age subgroup. However, no group difference in all-cause mortality was found between the RVOs and comparisons for each age subgroup. Previous studies revealed inconclusive findings. A population-based study in Korea showed that RVO was associated with an increased risk of stroke development in every age subgroup [14], whereas another administrative database study in Taiwan found a significant association that was present in only the 60- to 69-year subgroup [15]. The pooled data analysis of the Beaver Dam Eye Study and the Blue Mountain Eye Study revealed that RVO was not associated with stroke-related mortality in all age subgroups [23]. The inconsistencies of previous studies may have been due to heterogeneities in the inclusion/exclusion criteria, confounders for matching/adjustment, outcome definition, and follow-up period. With the largest sample size and the longest follow-up time to date, as well as a generally accepted coding system and matching/adjusting for possible confounders, our study provides more convincing results.

For the associations between RVO and stroke or all-cause mortality, very few studies have addressed the issue separately for the BRVO and CRVO groups at the same time [7, 12]. Although BRVO and CRVO are defined as venous occlusive diseases of the eye, they are not exactly the same in terms of their clinical manifestations, demographic characteristics, and prognostic factors [22, 26]. Thus, it is better to analyze them separately. In our study, among the 22,919 patients with RVO, 15,392 (67.2%) patients were diagnosed with BRVO and 7,527 (32.8%) were diagnosed with CRVO. The proportion was similar to that of a population-based study in the US [12]. As shown in Figure 2, both the BRVO and CRVO groups have a significantly higher risk of developing stroke, ischemic stroke, and hemorrhagic stroke than the comparison group after adjusting for PS. The previous Taiwan elderly population study conducted by Shih et al. and the Danish study conducted by Bertelsen et al. also revealed a significantly higher risk of stroke among both the BRVOs and CRVOs. However, a US study found a significantly higher risk of stroke only in the BRVO group [12]. The conflicts of previous studies may be attributed to the smaller sample size and, consequently, the larger CIs. Our study, with a larger sample size, can efficiently detect differences between groups. Another finding shown in Figure 2 is that after adjusting for PS, no group difference in all-cause mortality was found among the BRVOs, CRVOs, and their comparisons. This finding was similar to the results of most previous studies [3, 13, 16, 18]. This may be because of the treatment of systemic risk factors after the diagnosis of RVO.

One limitation of our study is that our inclusion of diagnoses was based on the claimed database, not on fundus photography and medical charts, as in hospital-based studies. However, in our database, the diagnoses of BRVO, CRVO, stroke, and confounding comorbidities were accurate and were verified by the National Health Administration (NHA). In our healthcare system, the NHA not only checks the consistencies between the claimed data and the charts but also ensures that the patient receives a standard protocol of examinations to confirm the diagnosis. Furthermore, in our database, we adopted the commonly used diagnosis classification system of ICD-9-CM codes. Thus, our results can be clearly interpreted and compared to further studies in other countries.

Another limitation was that some patients with asymptomatic RVO may not seek medication and may not be included in our RVO group. Thus, subjects with undetected RVO were classified as the comparison group and not the RVO group, which creates a misclassification bias. If RVO increasing the development of stroke is a real phenomenon, this bias will weaken the association between RVO and stroke. In our study, the association between RVO and stroke was significant, even in the presence of this bias. Therefore, the association will be stronger in real situations.

## 5. Conclusions

In our study, the RVO patients are at significantly greater risk of developing stroke, ischemic stroke, and hemorrhagic stroke. However, the risk of all-cause mortality was similar in RVOs and non-RVOs. Our study has clinical and public health implications. Clinically, our study suggests that ophthalmologists and patients should be aware of the possible increased risk of stroke. In particular, patients with RVO who have risk factors for stroke, such as diabetes and hypertension, should be referred to physicians for early diagnosis and treatment to prevent the occurrence of stroke. From a public health perspective, policy makers are encouraged to enforce surveillance to determine the stroke risks in patients with RVO.

## Figures and Tables

**Table 1:** Characteristics of the study subjects.

Variable	RVO group	Comparison group (real world)	*p*-value	Comparison group (PS-matched)	*p*-value
	*n* = 22919	*n* = 114595		*n* = 114595	
Age, year (mean±SD)	61.8 ± 13.0	43.2 ± 15.4	<0.0001	61.8 ± 13.0	>0.99

Age, categorical			<0.0001		>0.99
<50	3895 (17.0)	78047 (68.1)		19487 (17.0)	
50-60	5700 (24.9)	18802 (16.4)		28300 (24.9)	
60-70	6140 (26.8)	9819 (8.6)		30795 (26.8)	
>=70	7184 (31.3)	7927 (6.9)		36013 (31.3)	

Gender			0.04		0.99
Male	11801 (51.5)	58090 (50.7)		59015 (51.5)	
Female	11118 (48.5)	56505 (49.3)		55080 (48.5)	

Antithrombotic drugs	6437 (28.1)	8776 (7.7)	<0.0001	32175 (28.1)	0.99

Comorbidities					
Obesity	657 (2.9)	2388 (2.1)	<0.0001	3346 (2.9)	0.67
Diabetes	8635 (37.7)	14468 (12.6)	<0.0001	43090 (37.6)	0.84
Hypertension	17927 (78.2)	28935 (25.2)	<0.0001	89623 (78.2)	0.98
Hyperlipidemia	10991 (48.0)	22688 (19.8)	<0.0001	55005 (48.0)	0.91
Coronary artery disease	8173 (35.7)	12223 (10.7)	<0.0001	40909 (35.7)	0.92
Atrial fibrillation	973 (4.2)	1113 (1.0)	<0.0001	4809 (4.2)	0.75
Chronic kidney disease	2724 (11.9)	2468 (2.2)	<0.0001	13646 (11.9)	0.93
Hyperviscosity	252 (1.1)	344 (0.3)	<0.0001	1260 (1.1)	>0.99

CCI			<0.0001		>0.99
0	17017 (74.3)	105002 (91.6)		85150 (74.3)	
1	2668 (11.6)	5022 (4.4)		13300 (11.6)	
2	1673 (7.3)	2615 (2.3)		8353 (7.3)	
≥3	1561 (6.8)	1956 (1.7)		7792 (6.8)	

Glaucoma	2016 (8.8)	1718 (1.5)	<0.0001	10084 (8.8)	0.99

PS	0.11 ± 0.07			0.11 ± 0.07	1.00

Stroke					
Incident event	5481 (23.9)	7523 (6.6)	<0.0001	22235 (19.4)	<0.0001
Mean follow-up year	4.9 ± 3.5	6.2 ± 3.7	<0.0001	5.4 ± 3.5	<0.0001

Ischemic stroke					
Incident event	4867 (21.2)	6583 (5.7)	<0.0001	19742 (17.2)	<0.0001
Mean follow-up year	4.8 ± 3.5	6.2 ± 3.7	<0.0001	5.4 ± 3.5	<0.0001

Hemorrhagic stroke					
Incident event	813 (3.5)	1153 (1.0)	<0.0001	3063 (2.7)	<0.0001
Mean follow-up year	5.7 ± 3.5	6.4 ± 3.7	<0.0001	5.7 ± 3.6	0.54

All-cause mortality					
Incident event	3111 (13.6)	7972 (7.0)	<0.0001	15106 (13.2)	0.11
Mean follow-up year	5.8 ± 3.6	6.5 ± 3.7		5.8 ± 3.7	0.63

Data are presented in number (percentage) or mean ± SD. RVO = retinal vein occlusion; PS = propensity score; CCI = Charlson comorbidity index.

**Table 2:** Risk of the clinical outcomes in the RVO and comparison groups.

Clinical outcome	RVO group	Comparison group (PS-matched)	*p*-value
	*n* = 22919	*n* = 114595	
Stroke			
Incidence density	494.7	361.6	<0.0001
HR	1.37 (1.33–1.41)	Reference	<0.0001

Ischemic stroke			
Incidence density	430.6	317.0	<0.0001
HR	1.36 (1.32–1.40)	Reference	<0.0001

Hemorrhagic stroke			
Incidence density	62.2	46.6	<0.0001
HR	1.34 (1.24–1.44)	Reference	<0.0001

All-cause mortality			
Incidence density	233.7	226.6	0.11
HR	1.03 (0.99–1.07)	Reference	0.12

HR = hazard ratio; the unit of incidence density: per 10000 years.

**Table 3:** Comparison of the risk for the clinical outcomes between the RVO and PS-matched comparison groups, stratified by age subgropus.

Clinical outcome	Age < 50		50 ≤ age < 60		60 ≤ age < 70		Age ≥ 70	
	*n* = 23382		*n* = 34000		*n* = 36935		*n* = 43197	
	ID	HR	ID	HR	ID	HR	ID	HR
Stroke								
RVO	201.7	1.73 (1.56–1.93)	381.1	1.64 (1.53–1.75)	542.2	1.39 (1.32–1.47)	770.1	1.17 (1.12–1.23)
comparison	116.5	Reference	232.9	Reference	390.0	Reference	656.0	Reference

Ischemic stroke								
RVO	168.1	1.67 (1.49–1.88)	331.9	1.58 (1.47–1.69)	483.4	1.39 (1.32–1.48)	659.7	1.18 (1.12–1.24)
Comparison	100.6	Reference	210.6	Reference	346.9	Reference	559.8	Reference

Hemorrhagic stroke								
RVO	40.6	1.57 (1.25–1.99)	53.3	1.54 (1.30–1.82)	63	1.41 (1.22–1.63)	82.7	1.23 (1.09–1.40)
Comparison	25.8	Reference	34.7	Reference	44.7	Reference	67.2	Reference

All-cause mortality								
RVO	2	1.03 (0.89–1.19)	115.4	1.02 (0.92–1.14)	189.1	1.02 (0.94–1.10)	469.6	1.03 (0.98–1.09)
Comparison	85.7	Reference	113.1	Reference	186.2	Reference	455.5	Reference

ID = incidence density; HR = hazard ratio; the unit of Incidence density: per 10000-year.

## Data Availability

Data are available from the National Health Insurance Research Database (NHIRD) published by Taiwan National Health Insurance (NHI) Bureau. The data utilized in this study cannot be made available in the manuscript, the supplemental files, or in a public repository due to the “Personal Information Protection Act” executed by Taiwan's government, starting from 2012. Requests for data can be sent as a formal proposal to the NHIRD (http://nhird.nhri.org.tw) or by email to wt.gro.irhn@drihn.

## Abbreviations and Acronyms

RVO: Retinal vein occlusion
BRVO: Branch retinal vein occlusion
CRVO: Central retinal vein occlusion
HR: Hazard ratio
ICD-9-CM codes: International Classification of Diseases, Ninth Revision, Clinical Modification codes
KCD: Korean Classification of Diseases
NHI: National Health Insurance
NHIRD: National Health Insurance Research Database.
